# Sex Differences in the Patterns of Systemic Agent use Among Patients With Psoriasis: A Retrospective Cohort Study in Quebec, Canada

**DOI:** 10.3389/fphar.2022.810309

**Published:** 2022-02-15

**Authors:** Raymond Milan, Jacques LeLorier, Marie-Josée Brouillette, Anne Holbrook, Ivan V. Litvinov, Elham Rahme

**Affiliations:** ^1^ Department of Medicine, Division of Experimental Medicine, McGill University, Montreal, QC, Canada; ^2^ Centre for Outcomes Research and Evaluation, Research Institute of the McGill University Health Centre, Montreal, QC, Canada; ^3^ Faculté de Médecine, Université de Montréal, Montreal, QC, Canada; ^4^ Centre de Recherche Du Centre Hospitalier De L'Université de Montréal, Montreal, QC, Canada; ^5^ Department of Psychiatry, McGill University, Montreal, QC, Canada; ^6^ Department of Medicine, Division of Clinical Pharmacology and Toxicology, McMaster University, Hamilton, ON, Canada; ^7^ Department of Health Research Methods, Evidence and Impact, McMaster University, Hamilton, ON, Canada; ^8^ Research Institute of St. Joe’s Hamilton, St. Joseph’s Healthcare Hamilton, Hamilton, ON, Canada; ^9^ Department of Medicine, Division of Dermatology, McGill University, Montreal, QC, Canada; ^10^ Department of Medicine, Division of Clinical Epidemiology, McGill University, Montreal, QC, Canada

**Keywords:** sex, biologic agents, conventional systemic agents, treatment patterns, psoriasis

## Abstract

**Background:** Sex differences exist in psoriasis manifestation and expectations from treatment with systemic agents, including, conventional systemic agents (CSA) and tumor necrosis factor inhibitors or ustekinumab (TNFi/UST). However, sex differences in patterns of systemic agent use, such as CSA discontinuation and switch from CSA to TNFi/UST have not been examined.

**Objectives:** To assess sex differences in patterns of CSA use and identify factors associated with switch to (or add) a TNFi/UST and those associated with CSA discontinuation.

**Methods:** We conducted a retrospective cohort study using the Quebec health administrative databases. We included patients with psoriasis initiating a CSA in 2002–2015. We excluded patients with a psoriasis diagnosis in the 3 years prior to the first diagnosis date between 2002 and 2015, and those with a systemic agent dispensation in the year prior to that date. We used Cox regression models with the Least Absolute Shrinkage and Selection Operator method to identify factors associated with Switch/add TNFi/UST, and those associated with CSA discontinuation. Separate analyses were performed for male and female patients.

**Results:** We included 1,644 patients (55.7% females, mean age 60.3 years), among whom 60.4% discontinued their CSA and 7.4%, switched/added TNFi/UST (3.4% switched and 4.0% added) within a median of 0.78 years of follow-up. Among male and female patients, rates of Switch/add TNFi/UST per 1,000 person-year were 49.1 and 41.0 and rates of CSA discontinuation were 381.2 and 352.8. Clinical obesity in male patients (HR 3.53, 95% CI 1.20–10.35), and adjustment/somatoform/dissociative disorders (HR 3.17, 95% CI 1.28–7.85) and use of nonsteroidal anti-inflammatory drugs (HR 2.70, 95% CI 1.56–4.70) in female patients were associated with Switch/add TNFi/UST. Male patients followed by a rheumatologist (HR 0.66, 95% CI 0.46–0.94) and those with a prior hospitalization (HR 0.70, 95% CI 0.57–0.87) were at lower risk of CSA discontinuation, while those initiated on acitretin (vs methotrexate) were at higher risk to discontinue their CSA (HR 1.61, 95% CI 1.30–2.01). Female patients with rheumatoid arthritis comorbidity (HR 0.69, 95% CI 0.51–0.93), those with a dispensed lipid-lowering agent (HR 0.72, 95% CI 0.59–0.88) and hypoglycemic agent (HR 0.75, 95% CI 0.57–0.98) and those initiated on methotrexate (vs all other CSAs) were less likely to discontinue their CSA. Male and female patients entering the cohort between 2011 and 2015 were at reduced risk of CSA discontinuation compared to those entering the cohort before 2011.

**Conclusion:** Most male and female patients discontinued their CSA within 1 year of follow-up. Our study highlighted sex differences in patients’ characteristics associated with switch/add a TNFi/UST and CSA discontinuation; treatment switch and discontinuation may be indications of treatment failure in most patients.

## Introduction

Psoriasis is a chronic inflammatory skin condition affecting 1 to 3.2% of the population in western countries (Canadian Agency for Drugs and Technologies in Health, 2007; Rachakonda et al., 2014). About 21.5% of patients have a moderate-to-severe form of psoriasis (Canadian Dermatology Association, 2009; Armstrong et al., 2021). Psoriasis treatments vary by disease severity and include topical agents, phototherapy, conventional systemic agents (CSA) and biologic agents (Canadian Dermatology Association, 2009). Clinical guidelines for the management of psoriasis recommend treatment with systemic agents, including CSA and biologic agents, for moderate-to-severe psoriasis. Randomized controlled trials (RCT) have found biologic agents, including tumor necrosis factor alpha inhibitors (TNFi) and interleukin inhibitors, to be more effective than placebo and the CSA, methotrexate, in achieving complete or nearly complete skin clearance and maintaining it over a longer period of time in patients with moderate-to-severe psoriasis (Gordon et al., 2006; Mahil et al., 2020). An important barrier to biologic agents’ use is their high acquisition costs. The provincial drug plan in Quebec approves reimbursement for biologic agents in psoriasis only when CSA are contraindicated or ineffective (Régie de l’assurance maladie du Québec, 2020); a policy adopted by several other public drug insurance plans (Ighani et al., 2019; National Institute for Health and Care Excellence, 2020).

Although the prevalence of psoriasis is similar in male and female populations (Amur et al., 2012), sex differences in disease manifestation have been reported. Male and female patients with psoriasis differ in presence of comorbidities (Mahler et al., 2009; Love et al., 2011; Sondermann et al., 2020), and needs and goals from therapy (Uttjek et al., 2005; Mahler et al., 2009; Papp et al., 2010; Maul et al., 2019). Because male patients are at higher risk of moderate-to-severe psoriasis than female patients, they may be more likely to receive a systemic agent (biologics or CSA) (Hotard et al., 2000; White et al., 2012; Hagg et al., 2017). Female patients reported having higher treatment expectations and thus increased potential for perceived treatment failure and requests for treatment change (Generali et al., 2016; Maul et al., 2019). Differences in CSA prescribing and switching in male and female patients have not been clearly described (Generali et al., 2016). Switching and discontinuing treatment are indications of treatment failure and are associated with worsening of psoriasis severity, lower quality of life and psychiatric morbidity (Thorneloe et al., 2013; Michalek et al., 2016; Bell et al., 2020).

Most studies examining patterns of systemic agents’ use in psoriasis considered only those using biologic agents and examined treatment discontinuation and switch between these agents (No et al., 2018; Mourad et al., 2019). Little is known about the patterns of CSA use in this population (Tabolli et al., 2015; Higa et al., 2019). Our objectives were to assess among patients with psoriasis, sex differences in 1) the patterns of CSA use, 2) factors associated with switch/add a TNFi or ustekinumab (TNFi/UST), and 3) factors associated with CSA discontinuation.

## Patients and Methods

### Study Design and Data Source

We conducted a retrospective cohort study using the province of Quebec health administrative databases housed at the *Régie de l’assurance maladie du Québec* (RAMQ). Quebec has a universal health care system offering free of charge physician and hospital services to all residents. Drug insurance is mandatory since 1997. Individuals in the working force who do not have a private drug insurance plan with their employer, all those ≥65 years and all those receiving social assistance are registered in the public drug insurance plan. In 2015, 44.3% of all Quebec residents were covered by the provincial drug plan (Régie de l'Assurance Maladie du Québec, 2015). Socio-demographic, physician and prescription drug claims and hospital records were obtained from RAMQ for the period from January 1997 to December 2015. The pharmaceutical claims database contains information on prescribed medications, including dispensation date, dosage, duration of supply and prescriber specialty, for those covered by RAMQ drug plan. The medical claims database contains information on all outpatient physician claims for all Quebec residents (International Classification of Diseases 9th revision, ICD-9 codes). The hospital abstract records provide information on all hospital admissions including the admission/discharge dates and primary and secondary discharge diagnoses (ICD-9 codes before April 2006 and ICD-10 codes thereafter).

### Study Population

We selected individuals ages ≥20 years who received a first diagnostic code for psoriasis either in-hospital, during an emergency department (ED) or outpatient visit between January 2002 and September 2015 (ICD-9 code 696.1 and ICD-10 code L40.x). We considered those who were continuously enrolled in the provincial drug plan in the previous year and examined their treatment utilization until the first gap ≥90 days in their enrolment plan (eligibility period). We defined a new patient with psoriasis as one without any diagnosis code for psoriasis in the previous 3 years and any psoriasis treatment (phototherapy, CSA or a biologic agent) in the previous year. Among new patients, we included those initiating a CSA (methotrexate, cyclosporine, acitretin and sulfasalazine) anytime during their eligibility period. The date of the first CSA prescription fill was their index date. We excluded those who received any biologic agent in the prior year and those with less than 3 months of data following their index date. We also excluded those with a diagnosis of Human Immunodeficiency Virus (HIV), Hepatitis B Virus (HBV), active *tuberculosis*, congestive heart failure (CHF) and melanoma skin cancer in the prior 2 years because TNFi/UST are contraindicated in these conditions (Elmets et al., 2019; Enbrel® (Etanercept), 2000; Humira® (Adalimumab), 2004; Nardone et al., 2014; Remicade® (Infliximab), 2017; Simponi® (Golimumab), 2018; Stelara® (Ustekinumab), 2017).

### Outcomes

For this study, we considered all CSA as a single class. Our outcomes were 1) Switch/add TNFi/UST (etanercept, infliximab, adalimumab, golimumab, certolizumab pegol and ustekinumab); and 2) CSA discontinuation. These outcomes were determined based on the comparison between the time between the preceding CSA fill and the time to the next refill with the permissible treatment gap. For each CSA prescription fill, the duration of supply was available in the RAMQ pharmaceutical database. To the duration of supply, we added a 60-days grace period to compute the permissible gap. We defined Switch/add TNFi/UST as receiving a dispensed prescription for one of these agents within the permissible gap. An add-on occurred if the patient also refilled their CSA prescription within the permissible gap, while a switch occurred if they did not. We defined CSA discontinuation as no supply for any CSA for a period exceeding the permissible gap. We combined switch and add-on of TNFi/UST for statistical power purposes.

### Follow-Up

We followed the study individuals from index date until the first date of Switch/add TNFi/UST, CSA discontinuation, death, occurrence of an ineligibility criterion (dispensed prescription for a biologic agent not indicated for psoriasis, diagnosis for HIV, HBV, active *tuberculosis*, CHF and melanoma skin cancer), a gap ≥90 days in the provincial drug plan enrollment or 31 December 2015, whichever occurred first.

### Baseline Characteristics

We assessed the following potential predictors at baseline: *socio-demographic characteristics*: age, sex, area of residency (rural/urban), income (low/high based on receiving partial or total subsidies), social deprivation index (quintiles with 5 representing the most socially deprived); *all-cause healthcare use in the prior 2 years*: all-cause hospitalization and ED visits; *psoriasis treatment characteristics*: year of cohort entry (2002–2010 vs 2011–2015), psoriasis duration, specialty of the CSA prescriber, first CSA received, and use of topical agents and phototherapy in the prior year; *comorbidity in the prior 2 years* using ICD codes during at least one inpatient, outpatient or ED visit (whether treated or not): PSa, rheumatoid arthritis (RA), inflammatory bowel diseases (IBD), ankylosing spondylitis (AS), clinical obesity, hypertension, ischemic heart diseases, cerebrovascular diseases, vascular diseases, cardiac arrhythmias, renal diseases, liver diseases, respiratory diseases, cancer, mental health disorders and drug/alcohol abuse; and *medication use in the prior year* defined by having at least one dispensed prescription for the following drugs: antidepressants, benzodiazepines, opioids, antihypertensive agents, hypoglycemic agents, platelet inhibitors, anticoagulants, lipid-lowering agents, nonsteroidal anti-inflammatory drugs (NSAIDS) and oral corticosteroids.

### Statistical Analyses

We reported baseline characteristics by sex and compared them using univariable and multivariable logistic regression models. We conducted all remaining analyses in male and female patients separately. We reported crude incident rates (IR) of Switch/add TNFi/UST and CSA discontinuation per 1,000 person-years. We plotted Kaplan Meier curves for our primary outcomes by sex. We employed the Least Absolute Shrinkage and Selection Operator (LASSO) method to select potential baseline characteristics associated with Switch/add TNFi/UST and CSA discontinuation. LASSO method is applied to avoid overfitting by penalizing the absolute size of the regression coefficients with the L1-regularization penalty factor λ (Tibshirani, 1996; Kumar et al., 2019). By increasing the value of λ, non-influential baseline characteristics with weak estimates will shrink towards zero. The optimal λ was determined using a 10-fold cross validation (CV) and 500 iterations to reduce potential instability in the results. We selected the value of λ where the CV-error curve hits its minimum (Tibshirani, 1996; Simon et al., 2011; Kumar et al., 2019).

Before modelling, we assessed collinearity between binary variables using the tetrachoric correlation. If a pair was strongly correlated (r ≥ ±0.8), the most clinically relevant was selected. The remaining baseline characteristics were included in the Cox regression models with LASSO. Variables selected by LASSO were included in univariable Cox regression models. Those significant at the 0.25 level were included in a multivariable Cox regression model and standard backward variable selection procedures, based on a significance level of 0.05 and the Bayesian Information Criterion (BIC), were applied (Hosmer et al., 2008). The final Cox regression models for both sexes included all the variables selected in either the male and female models for each outcome. We verified the proportional hazard assumption using the Schoenfeld residuals. We assessed internal validity of the models by the Harrel’s Concordance index (discrimination measure) and calibration slopes (models’ reliability) (Harrell, 2011). We reported hazard ratios (HR) and 95% confidence intervals (CI).

In sensitivity analyses, to test the robustness of our main findings, we repeated the main analyses 1) in each of the age groups < 65 years and ≥65 years separately; 2), for each of the time periods defined by the year of cohort entry 2002–2010 and 2011–2015, separately. The analyses by time periods were conducted to control for a potential change in psoriasis management over time as TNFi and ustekinumab were included in the provincial drug formulary for psoriasis in late 2008 and 2011 respectively; before then, they were prescribed for psoriasis on the exceptional patient basis; 3) excluding patients with PSa at cohort entry; 4) considering only patients receiving their initial CSA from a dermatologist, rheumatologist, internal medicine specialist or a general practitioner; 5) not excluding patients with CHF because TNFi/UST are only contraindicated in patients with moderate-to-severe CHF (Remicade® (Infliximab), 2017; Simponi® (Golimumab), 2018; Humira® (Adalimumab), 2004); 6) varying the grace period from 60 to 30 and 90 days; and 7) excluding sulfasalazine from the list of CSA prescribed for psoriasis.

Cohort development and statistical analyses were performed using SAS (version 9.4) and R studio (version 3.6.2).

## Results

### Study Population

We included 1,644 patients with psoriasis who initiated a CSA (Supplementary eFigure S1), among whom 55.7% were females (Table 1). Most male (63.5%) and female (60.5%) patients were prescribed their first CSA by a dermatologist with methotrexate being the CSA most often prescribed (females: 58.5% and males: 56.0%) followed by acitretin, sulfasalazine and cyclosporine. Unadjusted and adjusted odds ratios and 95% CI are presented in Table 1. After adjusting for all baseline characteristics, compared to female patients, male patients were younger (mean age 58.6 ± 15.7 vs 61.4 ± 15.1 years), more likely to have higher income, ischemic heart diseases, cancer, renal diseases, and alcohol/drug abuse, and to use lipid-lowering drugs. Male patients were less likely to live in urban areas, to have vascular diseases, respiratory diseases, dissociative, somatoform and adjustment disorders and to use antidepressants and benzodiazepines.

**TABLE 1:** Baseline patient characteristics by sex: logistic regression model comparing baseline characteristics in male vs female patients.

	All patients (*N* = 1644)	Females (*N* = 916)	Males (*N* = 728)	Unadjusted OR (95% CI)		Adjusted OR (95% CI)^a^
Mean age (SD)	60.3 (15.4)	61.4 (15.1)	58.9 (15.7)	–		–
Mean duration of follow-up in years (SD)	1.66 (2.2)	1.70 (2.3)	1.59 (2.0)	–		–
Median duration of follow-up in years (Q1, Q3)	0.78 (0.39, 1.87)	0.76 (0.39, 2.01)	0.81 (0.38, 1 .77)	–		–
Socio-demographic variables, N (%)						
Age						
20–54 years	537 (32.7)	263 (28.7)	274 (37.6)		Ref	Ref
55–64 years	370 (22.5)	233 (25.4)	137 (18.8)		0.56 (0.43−0.74)	0.57 (0.43−0.77)
65–74 years	455 (27.7)	253 (27.6)	202 (27.7)		0.77 (0.60−0.99)	0.70 (0.52−0.95)
≥75 years	282 (17.2)	167 (18.2)	115 (15.8)		0.66 (0.49−0.89)	0.56 (0.39−0.82)
Social deprivation index						
Unknown	205 (12.5)	110 (12.0)	95 (13.0)		0.9 (0.62−1.31)	1.08 (0.72−1.63)
Most socially privileged	239 (14.5)	122 (13.3)	117 (16.1)		Ref	Ref
Privileged socially	251 (15.3)	150 (16.4)	101 (13.9)		0.70 (0.49−1.00)	0.70 (0.48−1.03)
Average socially deprivation	283 (17.2)	162 (17.7)	121 (16.6)		0.78 (0.55−1.10)	0.78 (0.54−1.13)
Deprived socially	328 (20.0)	173 (18.9)	155 (21.3)		0.93 (0.67−1.30)	1.07 (0.75−1.53)
Most socially deprived	338 (20.6)	199 (21.7)	139 (19.1)		0.73 (0.52−1.02)	0.90 (0.62−1.29)
Area of residency (urban vs rural)	1320 (80.3)	755 (82.4)	565 (77.6)		0.74 (0.58−0.94)	0.69 (0.52−0.90)
Income (Low vs high income)^b^	963 (58.6)	558 (60.9)	405 (55.6)		0.80 (0.66−0.98)	0.77 (0.62−0.96)
Variables related to CSA and other psoriasis treatments, N (%)						
Year of cohort entry (2011–2015 vs. 2002–2010)	740 (45.0)	421 (46.0)	319 (43.8)		0.92 (0.75−1.12)	0.81 (0.65−1.01)
Psoriasis duration^c^						
0–3 months	458 (27.9)	261 (28.5)	197 (27.1)		Ref	Ref
>3–12 months	315 (19.2)	185 (20.2)	130 (17.9)		0.93 (0.70−1.25)	0.92 (0.67−1.26)
>12 months	871 (53.0)	470 (51.3)	401 (55.1)		1.13 (0.90−1.42)	1.22 (0.95−1.57)
Specialty of the first CSA prescriber						
Dermatologist	1016 (61.8)	554 (60.5)	462 (63.5)		Ref	Ref
Rheumatologist	290 (17.6)	172 (18.8)	118 (16.2)		0.82 (0.63−1.07)	0.79 (0.53−1.17)
Others^d^	338 (20.6)	190 (20.7)	148 (20.3)		0.93 (0.73−1.20)	0.90 (0.66−1.22)
First CSA received						
Methotrexate	944 (57.4)	536 (58.5)	408 (56.0)		Ref	Ref
Cyclosporine	51 (3.1)	25 (2.7)	26 (3.6)		1.37 (0.78−2.40)	1.15 (0.62−2.15)
Acitretin	570 (34.7)	316 (34.5)	254 (34.9)		1.06 (0.86−1.30)	0.97 (0.76−1.25)
Sulfasalazine	79 (4.8)	39 (4.3)	40 (5.5)		1.35 (0.85−2.13)	1.42 (0.85−2.39)
Use of topical agents in the prior year	1389 (84.5)	775 (84.6)	614 (84.3)		0.98 (0.75−1.28)	0.93 (0.68−1.27)
Use of phototherapy in the prior year	206 (12.5)	111 (12.1)	95 (13.0)		1.09 (0.81−1.46)	1.09 (0.78−1.51)
All-cause health care use and comorbidities in the prior 2 years, N (%)						
Hospitalizations	552 (33.6)	311 (34.0)	241 (33.1)		0.96 (0.78−1.18)	0.88 (0.68−1.14)
Emergency department visits	899 (54.7)	496 (54.1)	403 (55.4)		1.05 (0.86−1.28)	1.13 (0.90−1.43)
Psoriatic arthritis	242 (14.7)	129 (14.1)	113 (15.5)		1.12 (0.85−1.47)	1.24 (0.90−1.70)
Rheumatoid arthritis	233 (14.2)	148 (16.2)	85 (11.7)		0.69 (0.52−0.91)	0.71 (0.50−1.02)
Inflammatory bowel diseases	27 (1.6)	14 (1.5)	13 (1.8)		1.17 (0.55−2.51)	1.02 (0.44−2.38)
Ankylosing spondylitis	23 (1.4)	13 (1.4)	10 (1.4)		0.97 (0.42−2.22)	1.34 (0.55−3.30)
Obesity	78 (4.7)	47 (5.1)	31 (4.3)		0.82 (0.52−1.31)	0.85 (0.50−1.44)
Hypertension	591 (35.9)	345 (37.7)	246 (33.8)		0.85 (0.69−1.04)	0.91 (0.68−1.20)
Ischemic heart diseases	95 (5.8)	43 (4.7)	52 (7.1)		1.56 (1.03−2.37)	1.66 (1.03−2.67)
Cerebrovascular diseases	41 (2.5)	19 (2.1)	22 (3.0)		1.47 (0.79−2.74)	1.37 (0.68−2.74)
Vascular diseases	139 (8.5)	89 (9.7)	50 (6.9)		0.69 (0.48−0.98)	0.59 (0.39−0.91)
Cardiac Arrhythmias	90 (5.5)	47 (5.1)	43 (5.9)		1.16 (0.76−1.78)	1.32 (0.81−2.16)
Renal diseases	51 (3.1)	18 (2.0)	33 (4.5)		2.37 (1.32−4.24)	4.09 (2.10−7.95)
Liver diseases	51 (3.1)	25 (2.7)	26 (3.6)		1.32 (0.76−2.31)	1.43 (0.76−2.68)
Respiratory diseases	311 (18.9)	196 (21.4)	115 (15.8)		0.69 (0.53−0.89)	0.61 (0.46−0.82)
Cancer^e^	190 (11.6)	95 (10.4)	95 (13.0)		1.30 (0.96−1.76)	1.56 (1.11−2.20)
Mental health disorders, N (%)						
No mental health disorder	1191 (72.4)	640 (69.9)	551 (75.7)		Ref	Ref
Anxiety and mood disorders	356 (21.7)	228 (24.9)	128 (17.6)		0.65 (0.51−0.83)	0.83 (0.62−1.12)
Dissociative, somatoform and adjustment disorders	28 (1.7)	21 (2.3)	7 (1.0)		0.39 (0.16−0.92)	0.34 (0.13−0.89)
Other mental health disorders	69 (4.2)	27 (2.9)	42 (5.8)		1.81 (1.10−2.97)	2.36 (1.37−4.05)
Drug and/or alcohol abuse	74 (4.5)	28 (3.1)	46 (6.3)		2.14 (1.32−3.46)	2.65 (1.53−4.59)
Drug use in the prior year, N (%)						
Antidepressants	382 (23.2)	258 (28.2)	124 (17.0)		0.52 (0.41−0.67)	0.56 (0.42−0.76)
Benzodiazepines	478 (29.1)	303 (33.1)	175 (24.0)		0.64 (0.51−0.80)	0.69 (0.54−0.90)
Opioids	351 (21.4)	200 (21.8)	151 (20.7)		0.94 (0.74−1.19)	1.11 (0.84−1.47)
Antihypertensive agents	793 (48.2)	451 (49.2)	342 (47.0)		0.91 (0.75−1.11)	0.93 (0.69−1.26)
Hypoglycemic agents	258 (15.7)	141 (15.4)	117 (16.1)		1.05 (0.81−1.38)	0.96 (0.69−1.32)
Lipid-lowering drugs	569 (34.6)	298 (32.5)	271 (37.2)		1.23 (1.00−1.51)	1.47 (1.13−1.93)
Platelet inhibitors	471 (28.6)	251 (27.4)	220 (30.2)		1.15 (0.93−1.42)	1.13 (0.85−1.51)
Anticoagulants	59 (3.6)	35 (3.8)	24 (3.3)		0.86 (0.51−1.46)	0.90 (0.48−1.69)
Nonsteroidal anti-inflammatory drugs	658 (40.0)	378 (41.3)	280 (38.5)		0.89 (0.73−1.09)	0.95 (0.75−1.21)
Oral corticosteroids	423 (25.7)	232 (25.3)	191 (26.2)		1.05 (0.84−1.31)	1.27 (0.97−1.66)

a The multivariable logistic regression model was adjusted for all variables included in Table 1. The reference group was female patients. OR >1 indicates higher likelihood among male patients. OR< 1 indicates lower likelihood among male patients.

b Income (high vs low) was based on the type of drug plan they had with those receiving partial or total subsidies classified as low income.

c Time from first psoriasis diagnosis until the first conventional systemic agent prescription fill.

d The others category included mostly general practitioners (63.9%) and internal medicine doctors (25.1%). Only 5 (1.8%) received their first CSA, from a gastroenterologist.

e 22 female patients and 21 male patients had non-melanoma skin cancer.

OR: odds ratios; CSA: conventional systemic agent; CI: confidence interval; Q1: first quartile; Q3: third quartile; ref: reference group; SD: standard deviation.

### Patterns of Systemic Agents’ use

Patients were followed for a median of 0.78 years (quartiles: 0.39 and 1.87). During the follow-up, 993 (60.4%) patients discontinued their treatment, and 121 patients (7.4%) had a Switch/add TNFi/UST; 56 (3.4%) switched to a TNFi/UST and 65 (4.0%) received a TNFi/UST as an add-on. The IRs per 1,000 person-years in male vs female patients were: Switch/add TNFi/UST 49.1 vs 41.0 and CSA discontinuation 381.2 vs 352.8 (Table 2). Among the 121 patients who had Switch/add TNFi/UST, most (92.6%) received a TNFi, specifically adalimumab and etanercept, in both sexes (Table 3). Nine patients (5 females and 4 males) received ustekinumab. Kaplan Meier curves exhibited a non-significant higher tendency to Switch/add TNFi/UST in male versus female patients (Supplementary eFigure S2).

**TABLE 2:** Crude rates of switch to a TNFi/UST or add-on and treatment discontinuation among male and female patients with psoriasis.

	All (*N* = 1,644)		Females (*N* = 916)		Males (*N* = 728)	
	Number of events	IR per 1000 person-years (95% CI)	Number of events	IR per 1000 person-years (95% CI)	Number of events	IR per 1000 person-years (95% CI)
Switch to TNFi/UST or add-on	121	44.5 (37.0−53.1)	64	41.0 (31.6−52.3)	57	49.1 (37.2−63.7)
Switch to TNFi/UST	56	20.6 (15.5−26.7)	27	17.3 (11.4−25.1)	29	25.0 (16.7−35.9)
Add-on of TNFi/UST	65	23.9 (18.4−30.4)	37	23.7 (16.7−32.7)	28	24.1 (16.0−34.9)
CSA discontinuation	993	364.9 (342.6−388.3)	551	352.8 (324.0−383.6)	442	381.2 (346.5−418.4)

a Per 1,000 person-years.

b All CSA, were considered as a single class medication, i. e., a switch between CSAs, was not considered.

c Individuals who did not refill their CSA, after receiving a prescription fill for a TNFi/UST, within the allowed treatment gap.

d Individuals who refilled their CSA, after receiving a prescription fill for a TNFi/UST, within the allowed treatment gap.

e Discontinuation defined as no supply for any CSA, or a biologic agent for a period exceeding the 60-days grace period.

CSA: conventional systemic agent; CI: confidence interval; IR: incident rate; TNFi: Tumor necrosis factor inhibitor; UST: ustekinumab.

**TABLE 3:** Tumor necrosis factor inhibitors and ustekinumab received after the switch/add-on during the follow-up.

	All patients *N* = 121 (%)	Females *N* = 64 (%)	Males *N* = 57 (%)
Tumor necrosis factor inhibitors	112 (92.6)	59 (92.2)	53 (93.0)
Adalimumab	52 (43.0)	27 (42.1)	25 (43.9)
Etanercept	34 (28.1)	17 (26.6)	17 (29.8)
Infliximab	17 (14.1)	9 (14.1)	8 (14.0)
Golimumab	8 (6.6)	5 (7.8)	3 (5.3)
Certolizumab pegol	1 (0.8)	1 (1.6)	0 (0)
Ustekinumab	9 (7.4)	5 (7.8)	4 (7.0)

### Variable Selection for the Models

In both sexes, hypertension and prior use of antihypertensive agents had a correlation coefficient r > 0.8. We chose to include hypertension in the model and not antihypertensive agents because of the possibility that these agents were prescribed for an indication other than hypertension. For the patient characteristics associated with Switch/add TNFi/UST in male patients, renal diseases, liver diseases and cancer were not considered in the model because 0 patients with these comorbidities switched/added TNFi/UST. For the female model, inflammatory bowel diseases and renal diseases were also not considered (0 switch/add-on in patients with these conditions as well). All variables were considered when examining factors associated with CSA discontinuation in both sexes.

### Factors Associated With Switch/Add TNFi/UST

In male patients, compared to those ages 20–54 years, patients ages 55–64 years, 65–74 years and ≥75 years were at lower risk of Switch/add TNFi/UST (HR = 0.26, 95% CI: 0.12–0.56; HR = 0.21, 95% CI: 0.09–0.45; and HR = 0.10, 95% CI: 0.02–0.40, respectively) while in female patients only those ages ≥75 years were at lower risk of Switch/add TNFi/UST (HR = 0.17, 95% CI: 0.04–0.71) (Table 4). Male patients with clinical obesity (HR = 3.53, 95% CI: 1.20–10.35) and duration of psoriasis >12 months vs 0–3 months (HR = 2.34, 95% CI: 1.09–5.03) were at higher risk of Switch/add TNFi/UST. Female patients, with (vs without) dissociative, somatoform and adjustment disorders were at higher risk of Switch/add TNFi/UST (HR = 3.17, 95% CI: 1.28-7.85). Female patients entering the cohort between 2011–2015 (vs 2002–2010) and those with prescribed NSAID use in the prior year were at higher risk (HR = 1.81, 95% CI: 1.05–3.12 and HR = 2.70, 95% CI: 1.56–4.70 respectively) while those with RA were at lower risk (HR = 0.41, 95% CI: 0.19–0.89) of Switch/add TNFi/UST.

**TABLE 4:** Predictors of switch to a TNFi/UST or add-on among males and female patients with psoriasis–Cox proportional Hazard models with LASSO.

	All patients (*N* = 1,644)	Females (*N* = 916)	Males (*N* = 728)
	aHR (95% CI)	aHR (95% CI)	aHR (95% CI)
Age			
20–54 years	Ref	Ref	Ref
55–64 years	0.50 (0.32−0.78)	0.80 (0.44−1.44)	0.26 (0.12−0.56)
65–74 years	0.36 (0.23−0.58)	0.61 (0.32−1.15)	0.21 (0.09−0.45)
≥75 years	0.12 (0.04−0.32)	0.17 (0.04−0.71)	0.10 (0.02−0.40)
Sex	1.15 (0.79−1.67)	–	–
cohort entry between 2011–2015	1.55 (1.04−2.31)	1.81 (1.05−3.12)	1.28 (0.71−2.30)
Psoriasis duration^a^			
0–2.99 months	Ref	Ref	Ref
3–12 months	0.61 (0.33−1.16)	0.48 (0.22−1.04)	0.93 (0.30−2.86)
>12 months	1.25 (0.80−1.95)	0.76 (0.43−1.36)	2.34 (1.09−5.03)
Rheumatoid arthritis	0.67 (0.40−1.12)	0.41 (0.19−0.89)	1.33 (0.63−2.82)
Clinical obesity	1.60 (0.76−3.33)	1.09 (0.38−3.12)	3.53 (1.20−10.35)
Mental health disorders			
No mental health disorder	Ref	Ref	Ref
Anxiety and mood disorders	1.24 (0.81−1.89)	1.06 (0.59−1.91)	1.54 (0.85−2.81)
Dissociative, somatoform and adjustment disorders	2.95 (1.24−7.01)	3.17 (1.28−7.85)	NA^b^
Other mental health disorders	1.51 (0.72−3.17)	1.58 (0.37−6.65)	1.45 (0.59−3.52)
Prior use of NSAIDS	1.87 (1.27−2.73)	2.70 (1.56−4.70)	1.04 (0.58−1.86)
Internal validity of the models			
Harrel’s C index (95% CI)	0.69 (0.66−0.72)	0.68 (0.64−0.72)	0.70 (0.61−0.78)
Calibration slope	0.90	0.62	0.81

a Time from first psoriasis diagnosis until the first conventional systemic agent prescription fill.

b In male patients, only seven patients had dissociative, somatoform and adjustment disorders; therefore, they were combined with had also anxiety and mood disorders.

aHR: adjusted Hazard ratios, CI: confidence intervals; Harrel’s C index: Harrel’s Concordance index; LASSO: least absolute shrinkage and selection operator; NSAIDS: Non-steroidal anti-inflammatory drugs; ref: reference group; TNFi: Tumor necrosis factor inhibitor; UST: Ustekinumab.

### Factors Associated With CSA Discontinuation

Male and female patients entering the cohort between 2011–2015 were at lower risk of CSA discontinuation when compared to those entering the cohort between 2002–2010 (females: HR = 0.78, 95% CI: 0.65–0.93; males: HR = 0.70, 95% CI: 0.57–0.86). Compared to patients receiving methotrexate as a first CSA, initiating acitretin in both sexes (females: HR = 2.07, 95% CI: 1.69–2.5; males: HR = 1.61, 95% CI: 1.30–2.01), and cyclosporine and sulfasalazine in female patients were associated with increased risks of CSA discontinuation (Table 5). Male patients prescribed their first CSA by a rheumatologist (vs dermatologist) and those with (vs without) prior hospitalizations were at lower risk of CSA discontinuation (HR = 0.66, 95% CI: 0.46–0.94 and HR = 0.70, 95% CI: 0.57–0.87 respectively). Female patients with RA, and those with prior use of hypoglycemic and lipid-lowering agents were at a decreased risk of discontinuation by at least 25%.

**TABLE 5:** Predictors of CSA discontinuation among males and female patients with psoriasis - Cox proportional Hazard models with LASSO.

	All patients (*N* = 1,644)	Females (*N* = 916)	Males (*N* = 728)
	aHR (95% CI)	aHR (95% CI)	aHR (95% CI)
Sex	0.99 (0.87−1.12)	–	–
cohort entry between 2011–2015	0.76 (0.66−0.86)	0.78 (0.65−0.93)	0.70 (0.57−0.86)
Specialty of the CSA prescriber			
Dermatologist	Ref	Ref	Ref
Rheumatologist	0.70 (0.56−0.89)	0.75 (0.55−1.01)	0.66 (0.46−0.94)
Other specialists	0.85 (0.71−1.03)	0.89 (0.69−1.14)	0.83 (0.62−1.10)
First CSA received			
Methotrexate	Ref	Ref	Ref
Cyclosporine	1.45 (1.01−2.08)	2.14 (1.30−3.55)	1.03 (0.62−1.73)
Acitretin	1.84 (1.59−2.13)	2.07 (1.69−2.53)	1.61 (1.30−2.01)
Sulfasalazine	1.44 (1.04−2.00)	1.56 (1.00−2.42)	1.31 (0.80−2.12)
Prior hospitalization	0.86 (0.75−0.99)	0.99 (0.82−1.19)	0.70 (0.57−0.87)
Rheumatoid arthritis	0.75 (0.60−0.94)	0.69 (0.51−0.93)	0.84 (0.59−1.19)
Prior use of Hypoglycemic agents	0.82 (0.68−1.00)	0.75 (0.57−0.98)	0.97 (0.72−1.29)
Prior use of lipid-lowering agents	0.78 (0.68−0.91)	0.72 (0.59−0.88)	0.90 (0.72−1.11)
Internal validity of the models			
Harrel’s C index (95% CI)	0.63 (0.61−0.65)	0.65 (0.59−0.70)	0.61 (0.59−0.64)
Calibration slope	0.94	0.95	0.79

List abbreviations: aHR: adjusted Hazard ratios, CI: confidence intervals; Harrel’s C index: Harrel’s Concordance index; LASSO: least absolute shrinkage and selection operator; ref: reference group; TNFi: Tumor necrosis factor inhibitor; UST: ustekinumab.

### Sensitivity Analyses

Overall, results of the sensitivity analyses were consistent with those of the main analyses (Supplementary eTables S1–S10), except for the two analyses including patients aged ≥65 years and those entering the cohort after 2011. None of the predictors for Switch/add TNFi/UST were significant among male and female patients aged ≥65 years (Supplementary eTable S2), most likely due to the smaller sample sizes (*n* = 737, *n* = 420 females and *n* = 317 males) and the lower risk of receiving these agents at this age as shown in our main analyses. Similarly, none of the predictors for Switch/add TNFi/UST were significant for patients entering the cohort after 2011 (Supplementary eTable S4), again perhaps due to the smaller sample sizes (*n* = 740, *n* = 421 females and *n* = 319 males). Nonetheless, it is worth noting that in both analyses, the direction of HR estimates remained the same as in the main analyses.

In the sensitivity analyses with grace periods of 30 and 90 days, female patients receiving their first CSA prescription by a rheumatologist were also at a reduced risk of CSA discontinuation (Supplementary eTable S8 and Supplementary eTable S9), while the result was borderline non-significant in the main analysis (HR = 0.75, 95% CI: 0.55–1.01).

## Discussion

To our knowledge, this is the first study to assess sex differences in factors associated with Switch/add TNFi/UST and CSA discontinuation among individuals with psoriasis who initiated a CSA. Despite the sex differences in baseline characteristics, there were no statistically significant differences in the rates of CSA discontinuation and Switch/add TNFi/UST among male and female patients. Nonetheless, most of the factors associated with these outcomes were sex specific. Factors associated with Switch/add TNFi/UST included younger age for both sexes, psoriasis duration and clinical obesity in male patients, and mental health disorders, RA and prior use of NSAIDS in female patients. Factors associated with CSA discontinuation included the CSA received at cohort entry, and cohort entry date after 2011 for both sexes, and the prescriber specialty and hospitalization in the prior year for male patients and RA, lipid-lowering and hypoglycemic agent use for female patients.

In our study, 7.4% of patients who initiated a CSA received a TNFi/UST in follow-up. A similar result was reported by a United States study where 6.3% of patients switched CSA treatment and 81.3% of them switched to a biologic agent (Higa et al., 2019). The decision to prescribe TNFi/UST for our psoriasis patients using CSA was not influenced by the physician specialty or the CSA received. Clinical obesity was associated with Switch/add TNFi/UST only in males. Obesity has been previously associated with increased psoriasis severity (Naldi et al., 2008), which may partially explain our result. However, it is not clear why this result was only observed in males. Previous studies also reported that older adults are less likely to receive biologic agents due to the increased risk of infections at this age (DeWitt et al., 2009; Geale et al., 2016).

Male patients with >12 months (vs ≤ 12 months) psoriasis duration were at higher risk of Switch/add TNFi/UST. Patients with longer disease durations may have had a more severe disease manifestation. To examine this possibility, we assessed the number of phototherapy sessions received in the previous 3 months. Among those with psoriasis duration >12 months, 11.2% received phototherapy (median 15; quartiles 9–24 sessions) compared to 7.1% of those with a disease duration of 0–3 months (median 5; quartiles 4–17 sessions).

Our female patients with dissociative, somatoform and adjustment disorders were more likely to Switch/add TNFi/UST. Over 30% of patients with psoriasis suffer from mental health disorders (Kotrulja et al., 2010; Ferreira et al., 2016; Wu et al., 2017), with higher prevalence observed among females (Wu et al., 2016; Duvetorp et al., 2020). TNFi/UST are prescribed on a weekly to 3 months basis, therefore, adherence with TNFi/UST may be improved over that of CSA (Osterberg & Blaschke, 2005). In addition, reduced risk of mental health disorders, fatigue and quality of life instead of mental health disorders and fatigue and quality of life have been reported among those treated with biologic agents during RCT (Tyring et al., 2006; Gooderham et al., 2016; Strober et al., 2018). Perceived better adherence and improved quality of life may explain the higher rate of switch among our female patients with mental health disorders. It is worth noting that prior opioids use was high in our cohort (21.4%), similar to what have been previously reported in individuals with moderate-to-severe psoriasis (Noe et al., 2020). However, prior opioid use was not significantly different between both sexes.

The presence of RA as a comorbidity in our female patients decreased the risk of switch/add, while the presence of PSa was not associated with switch/add. This was surprising as TNFi/UST are recommended in Psa and TNFi are recommended in RA (Menter et al., 2008).

Prior use of prescribed NSAIDs was associated with an increased risk of Switch/add TNFi/UST in our female patients. NSAIDs have analgesic and anti-inflammatory properties and are indicated for RA, AS, Psa and other arthropathies. Female patients with psoriasis using NSAIDs report less pain, less burning sensations and less depressive feelings (Maul et al., 2019), but may have higher expectations from CSA treatments which may explain their higher risk of switch (Maul et al., 2019).

In our study, methotrexate was the CSA most often prescribed in both sexes and was associated with less CSA discontinuation. A recent systematic review including 6 observational studies also reported high rates of CSA discontinuation in 1 year of follow-up (Mason et al., 2019). In this review, 50.3% of patients initiated on methotrexate remained persistent at 1 year vs 42.2 and 23.3% of patients initiated on acitretin and cyclosporine, respectively (Davila-Seijo et al., 2016; Mason et al., 2019). However, results from this review cannot be compared to ours as all included studies considered new and prevalent CSA users with only one study differentiating between these users in the analysis (Mason et al., 2019). We found only two published retrospective cohort studies that included first time CSA users (Bergqvist et al., 2019; Higa et al., 2019). Similar to our findings, these studies reported high rates of CSA discontinuation within 1 year of follow-up (≥75%) with acitretin having the highest rate, followed by cyclosporine and methotrexate (Bergqvist et al., 2019; Higa et al., 2019). Acitretin has a teratogenic effect and is contraindicated in young women (Canadian Dermatology Association, 2009). Nonetheless, in our study, discontinuation rate remained significant in the age stratified analyses for both sexes, which suggest that other reasons may be the cause.

Among male and female patients, the risk of CSA discontinuation was higher among those entering the cohort in 2011–2015 (vs 2002–2010), while the risk of Switch/add TNFi/UST was higher only among female patients entering the cohort in 2011–2015 (vs 2002–2010). This reflects the changes in the standard of care for patients with moderate-to-severe psoriasis in Quebec between 2008 and 2011 following the update of the Canadian clinical guideline for the management of plaque psoriasis in 2009 and the inclusion of TNFi and UST on the provincial drug formulary in late 2008 and 2011 respectively (Canadian Dermatology Association, 2009; Hagg et al., 2017).

In the sensitivity analyses with grace periods of 30 and 90 days, male and female patients who received their initial CSA from a rheumatologist (vs a dermatologist) were less likely to discontinue their CSA. Further investigation is needed to better understand the nature of this association because patients treated by a rheumatologist may have concomitant rheumatic manifestations.

Prior use of lipid-lowering and hypoglycemic agents were associated with lower risks of CSA discontinuation in our female patients. Similar to our study, dyslipidemia was associated with persistence to CSA in a previous study (Bergqvist et al., 2019). However, it is not clear why this association was only observed in female patients in our study. Differences in the metabolic syndromes risks and types have been reported between males and females with psoriasis. While higher risks of metabolic syndromes have been reported in female versus male patients, (Love et al., 2011; Danielsen et al., 2015; Sondermann et al., 2020), female patients in our study were more likely to receive hypoglycemic agents and male patients were more likely to receive lipid-lowering agents.

In our study, prior all-cause hospitalization was associated with a lower risk of CSA discontinuation among male patients. Hospitalization is an indicator of frailty and males may be at higher risk of psoriasis complications (Gordon et al., 2017; Zhang et al., 2018). Therefore, they may be more closely monitored which may have improved their adherence to therapy (Gordon et al., 2006; Zhang et al., 2018).

### Limitations

Our study has some limitations. First, our database does not include direct information on psoriasis severity. We have considered the use CSA as indication of moderate-to-severe psoriasis. Although this definition has been used by many authors and was previously validated (Egeberg et al., 2016; Executive Board, 133, 2013), it is not a gold standard and as such our study may have included some patients with mild psoriasis. Second, psoriasis types may be associated with TNFi/UST use. However, we were unable to distinguish the psoriasis type in our study because such information was not available in the database. Third, in our study, obesity may have been underestimated because it was based on clinical diagnoses which may mostly include morbid obesity. Fourth, because only 7.4% of our study patients switched/added TNFi/UST with about half of them switching and the other half adding the treatment, our analysis considered the combination of both outcomes to increase the statistical power. As predictors of switching from CSA to TNFi/UST may differ from those of adding TNFi/UST to CSA and information regarding these differences is lacking in the literature, our results should be interpreted with caution. To manage model overfitting and perform variable selection, we used LASSO regularization, a method widely used in several machine learning algorithms (Kumar et al., 2019; Tibshirani, 1996). Our models showed good overall performances with Harrel’s Concordance index and calibration slopes ≥0.6 (Table 4, Table 5). Fifth, while most of our patients have initiated on methotrexate, our analyses did not consider switch and add-on between CSA. Sixth, over-the-counter pain-relief medicines are not included in the RAMQ pharmaceutical claims database. Therefore, we may have misclassified users of the over-the-counter NSAIDS as non-users which may have biased our results toward the null. The effect of NSAIDS use on Switch/add TNFi/UST may have been stronger than that reported in our study (Carrasco-Garrido et al., 2010). Seventh, our study did not include newer generations of biologic agents approved after 2015. Therefore, our results may not be generalizable to all biologic agents. Eighth, our results may not be generalizable to patients covered by private drug insurance plans. However, individuals from different socioeconomic statuses are covered by the RAMQ drug plan and in our study the variable income, based on the type of drug coverage with RAMQ, was not associated with both outcomes (Régie de l'Assurance Maladie du Québec, 2015). Lastly, our study is observational in nature and may suffer from residual confounding due to unmeasured confounders such as body mass index, pain, and smoking.

## Conclusion

In our study, a high proportion of male and female patients with psoriasis discontinued their CSA within the first year of initiating their systemic treatment. Our findings suggest that factors associated with Switch/add TNFi/UST include mostly characteristics related to patients’ clinical profile such as mental health disorders in female, and clinical obesity and disease duration in male patients. However, CSA discontinuation among male and female patients was also influenced by the initial CSA received and the speciality of the prescriber. Additional studies examining sex differences in systemic agents’ use are needed to confirm our findings and their impact on clinical practice and provincial drug policy. The identification of such factors may help improve the management of male and female patients with moderate-to-severe psoriasis when initiating systemic agents.

## Data Availability

The original contributions presented in the study are included in the article/Supplementary Material, further inquiries can be directed to the corresponding author.
