# Supplementary material for: Sex Differences in the Patterns of Systemic Agent use Among Patients With Psoriasis: A Retrospective Cohort Study in Quebec, Canada
Source: Front Pharmacol. 2022 Feb 15;13:810309. doi: 10.3389/fphar.2022.810309 (PMC8886891; doi:10.3389/fphar.2022.810309)
Supplement: Supplementary file 1 [file DataSheet1.docx]

SUPPLEMENT ELECTRONIC MATERIALS

[eTable 1: Sensitivity analysis – Predictors of switch to a TNFi/UST or add-on and CSA discontinuation among males and females with psoriasis – Patients < 65 years (N=907) 2](#_Toc90740102)

[eTable 2: Sensitivity analysis – Predictors of switch to a TNFi/UST or add-on and CSA discontinuation among males and females with psoriasis –Patients ≥ 65 years (N=737) 3](#_Toc90740103)

[eTable 3: Sensitivity analysis – Predictors of switch to a TNFi/UST or add-on and CSA discontinuation among males and females with psoriasis – Patients entering the cohort between January 01, 2002 until December 31, 2010 (N=904) 4](#_Toc90740104)

[eTable 4: Sensitivity analysis – Predictors of switch to a TNFi/UST or add-on and CSA discontinuation among males and females with psoriasis – Patients entering the cohort between January 01, 2011 until December 31, 2015 (N=740) 5](#_Toc90740105)

[eTable 5: Sensitivity analysis – Predictors of switch to a TNFi/UST or add-on and CSA discontinuation among males and females with psoriasis – Patients without psoriatic arthritis at baseline (N=1,402) 6](#_Toc90740106)

[eTable 6: Sensitivity analysis – Predictors of switch to a TNFi/UST or add-on and CSA discontinuation among males and females with psoriasis – Patients receiving their initial CSA from a dermatologist, rheumatologist, internal medicine specialist or a general practitioner (N=1,607) 7](#_Toc90740107)

[eTable 7: Sensitivity analysis – Predictors of switch to a TNFi/UST or add-on and CSA discontinuation among males and females with psoriasis – Without excluding patients with congestive heart failure (N=1,755) 8](#_Toc90740108)

[eTable 8: Sensitivity analysis – Predictors of switch to a TNFi/UST or add-on and CSA discontinuation among males and females with psoriasis – Grace period of 30 days (N=1,644) 9](#_Toc90740109)

[eTable 9: Sensitivity analysis – Predictors of switch to a TNFi/UST or add-on and CSA discontinuation among males and females with psoriasis – Grace period of 90 days (N=1,644) 10](#_Toc90740110)

[eTable 10: Sensitivity analysis – Predictors of switch to a biologic agent and CSA discontinuation among males and females with psoriasis – Without considering sulfasalazine as a CSA (N=1,610) 11](#_Toc90740111)

[eFigure 1: Study flowchart 12](#_Toc90740112)

[eFigure 2: Kaplan Meier estimates of switch to (a) TNFi/UST or add-on; and (b) CSA discontinuation by sex 13](#_Toc90740113)

# **eTable 1: Sensitivity analysis – Predictors of switch to a TNFi/UST or add-on and CSA discontinuation among males and females with psoriasis – Patients < 65 years (N=907)**

|  | Switch a TNFi/UST or add-on | | CSA discontinuation | |
| --- | --- | --- | --- | --- |
|  | Females (N=496) | Males (N=411) | Females (N=496) | Males (N=411) |
| Number of events | 46 | 47 | 249 | 249 |
|  | aHR (95% CI) | aHR (95% CI) | aHR (95% CI) | aHR (95% CI) |
| Cohort entry after 2011 | 1.53 [0.79, 2.95] | 1.30 [0.69, 2.46] | 0.85 [0.66, 1.08] | 0.56 [0.42, 0.74] |
| Age |  |  |  |  |
| 20-54 years | Ref | Ref | – | – |
| 55-64 years | 0.79 [0.44, 1.44] | 0.27 [0.12, 0.57] |  |  |
| Time to first CSA prescription |  |  |  |  |
| 0–2.99 months | Ref | Ref | – | – |
| 3­-12 months | 0.52 [0.21, 1.30] | 0.83 [0.24, 2.89] |  |  |
| >12 months | 0.81 [0.41, 1.61] | 2.09 [0.91, 4.80] |  |  |
| Specialty of the CSA prescriber |  |  |  |  |
| Dermatologist | – | – | Ref | Ref |
| Rheumatologist |  |  | 0.83 [0.55, 1.25] | 0.58 [0.35, 0.97] |
| Other specialists |  |  | 0.85 [0.60, 1.19] | 0.77 [0.52, 1.14] |
| First CSA received |  |  |  |  |
| Methotrexate | – | – | Ref | Ref |
| Cyclosporine |  |  | 1.79 [0.97, 3.29] | 1.05 [0.59, 1.86] |
| Acitretin |  |  | 2.13 [1.61, 2.82] | 1.75 [1.31, 2.34] |
| Sulfasalazine |  |  | 1.47 [0.81, 2.65] | 0.77 [0.33, 1.81] |
| Prior hospitalization | – | – | 1.11 [0.86, 1.43] | 0.68 [0.49, 0.93] |
| Rheumatoid arthritis | 0.39 [0.15, 1.04] | 1.49 [0.63, 3.53] | 0.78 [0.52, 1.18] | 0.41 [0.20, 0.82] |
| Obesity | 1.36 [0.46, 4.02] | 5.89 [1.90, 18.27] | – | – |
| Mental health disorders |  |  |  |  |
| No mental health disorder | Ref | Ref | – | – |
| Anxiety and mood disorders | 1.02 [0.51, 2.02] | 1.54 [0.80, 2.97] |  |  |
| Dissociative, somatoform and adjustment disorders | 3.88 [1.40, 10.72] | NA^a^ |  |  |
| Other mental health disorders | 1.06 [0.14, 8.15] | 2.02 [0.79, 5.12] |  |  |
| Prior use of Hypoglycemic agents | – | – | 0.72 [0.49, 1.07] | 0.81 [0.51, 1.30] |
| Prior use of lipid-lowering agents | – | – | 0.68 [0.51, 0.92] | 0.84 [0.60, 1.16] |
| Prior use of NSAIDS | 3.53 [1.75, 7.09] | 1.05 [0.54, 2.04] | – | – |

^a^In male patients, only 4 patients had dissociative, somatoform and adjustment disorders; therefore, they were combined with had also anxiety and mood disorders

List of abbreviations: aHR: Adjusted hazard ratios; CI: confidence interval; NSAIDS: nonsteroidal anti-inflammatory drugs

# **eTable 2: Sensitivity analysis – Predictors of switch to a TNFi/UST or add-on and CSA discontinuation among males and females with psoriasis –Patients ≥ 65 years (N=737)**

|  | Switch a TNFi/UST or add-on | | CSA discontinuation | |
| --- | --- | --- | --- | --- |
|  | Females (N=420) | Males (N=317) | Females (N=420) | Males (N=317) |
| Number of events | 18 | 10 | 242 | 193 |
|  | aHR (95% CI) | aHR (95% CI) | aHR (95% CI) | aHR (95% CI) |
| Cohort entry after 2011 | 2.96 [1.01, 8.70] | 1.08 [0.24, 4.86] | 0.69 [0.53, 0.90] | 0.86 [0.63, 1.17] |
| Age |  |  |  |  |
| 65-70 years | Ref | Ref | – | – |
| ≥75 years | 0.24 [0.05, 1.08] | 0.48 [0.10, 2.30] |  |  |
| Time to first CSA prescription |  |  |  |  |
| 0–2.99 months | Ref | Ref | – | – |
| 3­-12 months | 0.67 [0.15, 3.08] | 1.30 [0.08, 21.34] |  |  |
| >12 months | 1.08 [0.32, 3.60] | 4.04 [0.49, 33.03] |  |  |
| Specialty of the CSA prescriber |  |  |  |  |
| Dermatologist | – | – | Ref | Ref |
| Rheumatologist |  |  | 0.66 [0.42, 1.05] | 0.67 [0.39, 1.12] |
| Other specialists |  |  | 0.94 [0.64, 1.37] | 0.84 [0.54, 1.31] |
| First CSA received |  |  |  |  |
| Methotrexate | – | – | Ref | Ref |
| Cyclosporine |  |  | 4.69 [1.86, 11.79] | 1.04 [0.25, 4.30] |
| Acitretin |  |  | 2.01 [1.50, 2.70] | 1.44 [1.02, 2.04] |
| Sulfasalazine |  |  | 1.82 [0.93, 3.56] | 2.17 [1.17, 4.02] |
| Prior hospitalization | – | – | 0.87 [0.66, 1.14] | 0.76 [0.56, 1.04] |
| Rheumatoid arthritis | 0.50 [0.13, 1.87] | 0.93 [0.18, 4.89] | 0.60 [0.39, 0.93] | 1.24 [0.77, 1.97] |
| Obesity | NA | NA | – | – |
| Mental health disorders |  |  |  |  |
| No mental health disorder | Ref | Ref | – | – |
| Anxiety and mood disorders | 1.72 [0.52, 5.63] | 1.24 [0.25, 6.20] |  |  |
| Dissociative, somatoform and adjustment disorders | 1.71 [0.20, 14.47] | NA^a^ |  |  |
| Other mental health disorders | 1.76 [0.21, 14.48] | NA |  |  |
| Prior use of Hypoglycemic agents | – | – | 0.80 [0.55, 1.16] | 1.14 [0.78, 1.68] |
| Prior use of lipid-lowering agents | – | – | 0.75 [0.57, 0.98] | 0.83 [0.61, 1.13] |
| Prior use of NSAIDS | 0.92 [0.24, 3.51] | 0.92 [0.24, 3.51] | – | – |

^a^In male patients, only 3 patients had dissociative, somatoform and adjustment disorders; therefore, they were combined with had also anxiety and mood disorders

List of abbreviations: aHR: Adjusted hazard ratios; CI: confidence interval; NSAIDS: nonsteroidal anti-inflammatory drugs

# **eTable 3: Sensitivity analysis – Predictors of switch to a TNFi/UST or add-on and CSA discontinuation among males and females with psoriasis – Patients entering the cohort between January 01, 2002 until December 31, 2010 (N=904)**

|  | Switch a TNFi/UST or add-on | | CSA discontinuation | |
| --- | --- | --- | --- | --- |
|  | Females (N=495) | Males (N=409) | Females (N=495) | Males (N=409) |
| Number of events | 34 | 35 | 352 | 291 |
|  | aHR (95% CI) | aHR (95% CI) | aHR (95% CI) | aHR (95% CI) |
| Age |  |  |  |  |
| 20-54 years | Ref | Ref | – | – |
| 55-64 years | 0.83 [0.37, 1.84] | 0.20 [0.07, 0.55] |  |  |
| 65-74 years | 0.33 [0.10, 1.07] | 0.19 [0.07, 0.52] |  |  |
| ≥75 years | 0.28 [0.06, 1.30] | 0.17 [0.04, 0.74] |  |  |
| Time to first CSA prescription |  |  |  |  |
| 0–2.99 months | Ref | Ref | – | – |
| 3­-12 months | 0.57 [0.21, 1.59] | 0.85 [0.15, 4.71] |  |  |
| >12 months | 0.8 [0.35, 1.84] | 3.84 [1.30, 11.35] |  |  |
| Specialty of the CSA prescriber |  |  |  |  |
| Dermatologist | – | – | Ref | Ref |
| Rheumatologist |  |  | 0.71 [0.5, 0.90] | 0.65 [0.43, 1.00] |
| Other specialists |  |  | 0.88 [0.65, 1.20] | 0.70 [0.49, 1.01] |
| First CSA received |  |  |  |  |
| Methotrexate | – | – | Ref | Ref |
| Cyclosporine |  |  | 3.87 [2.01, 7.47] | 0.87 [0.46, 1.62] |
| Acitretin |  |  | 1.97 [1.53, 2.54] | 1.60 [1.21, 2.10] |
| Sulfasalazine |  |  | 1.34 [0.79, 2.29] | 1.17 [0.64, 2.14] |
| Prior hospitalization | – | – | 1.00 [0.79, 1.26] | 0.67 [0.51, 0.88] |
| Rheumatoid arthritis | 0.25 [0.07, 0.88] | 1.18 [0.48, 2.92] | 0.71 [0.50, 1.01] | 0.82 [0.55, 1.22] |
| Obesity | 0.98 [0.21, 4.50] | 9.23 [1.88, 45.26] | – | – |
| Mental health disorders |  |  |  |  |
| No mental health disorder | Ref | Ref | – | – |
| Anxiety and mood disorders | 1.30 [0.60, 2.83] | 1.28 [0.58, 2.84] |  |  |
| Dissociative, somatoform and adjustment disorders | 12.25 [3.38, 44.36] | NA^a^ |  |  |
| Other mental health disorders | 1.47 [0.19, 11.66] | 1.64 [0.47, 5.67] |  |  |
| Prior use of Hypoglycemic agents | – | – | 0.66 [0.47, 0.93] | 0.91 [0.61, 1.36] |
| Prior use of lipid-lowering agents | – | – | 0.76 [0.59, 0.96] | 0.93 [0.71, 1.21] |
| Prior use of NSAIDS | 3.29 [0.79, 7.82] | 0.88 [0.42, 1.87] | – | – |

^a^In male patients, only 5 patients had dissociative, somatoform and adjustment disorders; therefore, they were combined with had also anxiety and mood disorders

List of abbreviations: aHR: Adjusted hazard ratios; CI: confidence interval; NSAIDS: nonsteroidal anti-inflammatory drugs

# **eTable 4: Sensitivity analysis – Predictors of switch to a TNFi/UST or add-on and CSA discontinuation among males and females with psoriasis – Patients entering the cohort between January 01, 2011 until December 31, 2015 (N=740)**

|  | Switch a TNFi/UST or add-on | | CSA discontinuation | |
| --- | --- | --- | --- | --- |
|  | Females (N=421) | Males (N=319) | Females (N=421) | Males (N=319) |
| Number of events | 30 | 22 | 199 | 151 |
|  | aHR (95% CI) | aHR (95% CI) | aHR (95% CI) | aHR (95% CI) |
| Age |  |  |  |  |
| 20-54 years | Ref | Ref | – | – |
| 55-64 years | 0.93 [0.36, 2.39] | 0.39 [0.12, 1.25] |  |  |
| 65-74 years | 0.98 [0.41, 2.32] | 0.22 [0.06, 0.78] |  |  |
| ≥75 years | NA | NA |  |  |
| Time to first CSA prescription |  |  |  |  |
| 0–2.99 months | Ref | Ref | – | – |
| 3­-12 months | 0.50 [0.15, 1.69] | 1.06 [0.23, 4.97] |  |  |
| >12 months | 0.81 [0.34, 1.91] | 1.62 [0.52, 5.08] |  |  |
| Specialty of the CSA prescriber |  |  |  |  |
| Dermatologist | – | – | Ref | Ref |
| Rheumatologist |  |  | 0.89 [0.49, 1.62] | 0.63 [0.32, 1.24] |
| Other specialists |  |  | 0.90 [0.58, 1.40] | 1.08 [0.68, 1.72] |
| First CSA received |  |  |  |  |
| Methotrexate | – | – | Ref | Ref |
| Cyclosporine |  |  | 1.32 [0.60, 2.89] | 1.79 [0.71, 4.54] |
| Acitretin |  |  | 2.17 [1.56, 3.03] | 1.64 [1.13, 2.39] |
| Sulfasalazine |  |  | 2.40 [1.08, 5.35] | 1.66 [0.73, 3.79] |
| Prior hospitalization | – | – | 0.93 [0.69, 1.26] | 0.78 [0.55, 1.06] |
| Rheumatoid arthritis | 0.68 [0.25, 1.85] | 1.78 [0.44, 7.27] | 0.60 [0.34, 1.09] | 1.09 [0.50, 2.36] |
| Obesity | 1.03 [0.23, 4.67] | 2.08 [0.44, 9.77] | – | – |
| Mental health disorders |  |  |  |  |
| No mental health disorder | Ref | Ref | – | – |
| Anxiety and mood disorders | 0.93 [0.36, 2.43] | 2.04 [0.76, 5.47] |  |  |
| Dissociative, somatoform and adjustment disorders | 1.13 [0.26, 5.01] | NA^a^ |  |  |
| Other mental health disorders | 1.43 [0.18, 11.57] | 1.55 [0.39, 6.15] |  |  |
| Prior use of Hypoglycemic agents | – | – | 0.95 [0.61, 1.48] | 1.05 [0.68, 1.64] |
| Prior use of lipid-lowering agents | – | – | 0.65 [0.46, 0.92] | 0.85 [0.59, 1.23] |
| Prior use of NSAIDS | 2.20 [1.03, 4.73] | 1.24 [0.47, 3.25] | – | – |

^a^In male patients, only 2 patients had dissociative, somatoform and adjustment disorders; therefore, they were combined with had also anxiety and mood disorders

List of abbreviations: aHR: Adjusted hazard ratios; CI: confidence interval; NSAIDS: nonsteroidal anti-inflammatory drugs

# **eTable 5: Sensitivity analysis – Predictors of switch to a TNFi/UST or add-on and CSA discontinuation among males and females with psoriasis – Patients without psoriatic arthritis at baseline (N=1,402)**

|  | Switch a TNFi/UST or add-on | | CSA discontinuation | |
| --- | --- | --- | --- | --- |
|  | Females (N=787) | Males (N=615) | Females (N=787) | Males (N=615) |
| Number of events | 47 | 41 | 493 | 391 |
|  | aHR (95% CI) | aHR (95% CI) | aHR (95% CI) | aHR (95% CI) |
| Cohort entry after 2011 | 2.29 [1.21, 4.32] | 1.32 [0.64, 2.69] | 0.75 [0.62, 0.91] | 0.72 [0.58, 0.90] |
| Age |  |  |  |  |
| 20-54 years | Ref | Ref | – | – |
| 55-64 years | 0.63 [0.32, 1.26] | 0.30 [0.13, 0.68] |  |  |
| 65-74 years | 0.57 [0.27, 1.20] | 0.14 [0.05, 0.41] |  |  |
| ≥75 years | NA | 0.14 [0.03, 0.61] |  |  |
| Time to first CSA prescription |  |  |  |  |
| 0–2.99 months | Ref | Ref | – | – |
| 3­-12 months | 0.24 [0.08, 0.73] | 1.27 [0.38, 4.30] |  |  |
| >12 months | 0.81 [0.42, 1.56] | 2.10 [0.85, 5.18] |  |  |
| Specialty of the CSA prescriber |  |  |  |  |
| Dermatologist | – | – | Ref | Ref |
| Rheumatologist |  |  | 0.79 [0.56, 1.13] | 0.77 [0.50, 1.20] |
| Other specialists |  |  | 0.96 [0.73, 1.24] | 0.86 [0.64, 1.16] |
| First CSA received |  |  |  |  |
| Methotrexate | – | – | Ref | Ref |
| Cyclosporine |  |  | 2.31 [1.39, 3.83] | 1.20 [0.71, 2.02] |
| Acitretin |  |  | 2.10 [1.71, 2.59] | 1.57 [1.25, 1.98] |
| Sulfasalazine |  |  | 1.31 [0.78, 2.21] | 1.65 [0.95, 2.85] |
| Prior hospitalization | – | – | 1.03 [0.85, 1.26] | 0.65 [0.51, 0.81] |
| Rheumatoid arthritis | 0.64 [0.27, 1.51] | 1.23 [0.52, 2.93] | 0.64 [0.46, 0.90] | 0.68 [0.45, 1.02] |
| Obesity | 1.61 [0.54, 4.79] | 3.76 [0.85, 16.62] | – | – |
| Mental health disorders |  |  |  |  |
| No mental health disorder | Ref | Ref | – | – |
| Anxiety and mood disorders | 1.02 [0.50, 2.07] | 1.71 [0.84, 3.49] |  |  |
| Dissociative, somatoform and adjustment disorders | 4.23 [1.51, 11.80] | NA^a^ |  |  |
| Other mental health disorders | 2.03 [0.47, 8.74] | 1.75 [0.64, 4.80] |  |  |
| Prior use of Hypoglycemic agents | – | – | 0.71 [0.53, 0.95] | 0.90 [0.66, 1.23] |
| Prior use of lipid-lowering agents | – | – | 0.75 [0.61, 0.92] | 0.94 [0.75, 1.18] |
| Prior use of NSAIDS | 2.93 [1.55, 5.53] | 1.23 [0.61, 2.49] | – | – |

^a^In male patients, only 7 patients had dissociative, somatoform and adjustment disorders; therefore, they were combined with had also anxiety and mood disorders

List of abbreviations: aHR: Adjusted hazard ratios; CI: confidence interval; NSAIDS: nonsteroidal anti-inflammatory drugs

# **eTable 6: Sensitivity analysis – Predictors of switch to a TNFi/UST or add-on and CSA discontinuation among males and females with psoriasis – Patients receiving their initial CSA from a dermatologist, rheumatologist, internal medicine specialist or a general practitioner (N=1,607)**

|  | Switch a TNFi/UST or add-on | | CSA discontinuation | |
| --- | --- | --- | --- | --- |
|  | Females (N=898) | Males (N=709) | Females (N=898) | Males (N=709) |
| Number of events | 63 | 55 | 538 | 432 |
|  | aHR (95% CI) | aHR (95% CI) | aHR (95% CI) | aHR (95% CI) |
| Cohort entry after 2011 | 1.82 [1.05, 3.14] | 1.44 [0.80, 2.60] | 0.79 [0.66, 0.95] | 0.70 [0.57, 0.86] |
| Age |  |  |  |  |
| 20-54 years | Ref | Ref | – | – |
| 55-64 years | 0.75 [0.42, 1.37] | 0.26 [0.12, 0.57] |  |  |
| 65-74 years | 0.59 [0.31, 1.12] | 0.21 [0.09, 0.45] |  |  |
| ≥75 years | 0.16 [0.04, 0.69] | 0.09 [0.02, 0.39] |  |  |
| Time to first CSA prescription |  |  |  |  |
| 0–2.99 months | Ref | Ref | – | – |
| 3­-12 months | 0.51 [0.23, 1.11] | 0.85 [0.28, 2.63] |  |  |
| >12 months | 0.80 [0.44, 1.45] | 2.21 [1.03, 4.75] |  |  |
| Specialty of the CSA prescriber |  |  |  |  |
| Dermatologist | – | – | Ref | Ref |
| Rheumatologist |  |  | 0.74 [0.54, 1.01] | 0.67 [0.46, 0.96] |
| Other specialists |  |  | 0.83 [0.64, 1.08] | 0.86 [0.64, 1.16] |
| First CSA received |  |  |  |  |
| Methotrexate | – | – | Ref | Ref |
| Cyclosporine |  |  | 1.88 [1.08, 3.26] | 1.11 [0.64, 1.93] |
| Acitretin |  |  | 2.05 [1.67, 2.50] | 1.65 [1.32, 2.07] |
| Sulfasalazine |  |  | 1.70 [1.08, 2.67] | 1.37 [0.82, 2.30] |
| Prior hospitalization | – | – | 0.98 [0.81, 1.18] | 0.71 [0.57, 0.88] |
| Rheumatoid arthritis | 0.42 [0.19, 0.90] | 1.31 [0.61, 2.83] | 0.67 [0.49, 0.91] | 0.84 [0.58, 1.20] |
| Obesity | 1.13 [0.39, 3.25] | 2.62 [0.77, 8.85] | – | – |
| Mental health disorders |  |  |  |  |
| No mental health disorder | Ref | Ref | – | – |
| Anxiety and mood disorders | 1.00 [0.55, 1.83] | 1.55 [0.83, 2.87] |  |  |
| Dissociative, somatoform and adjustment disorders | 3.09 [1.25, 7.64] | NA^a^ |  |  |
| Other mental health disorders | 1.56 [0.37, 6.56] | 1.88 [0.78, 4.55] |  |  |
| Prior use of Hypoglycemic agents | – | – | 0.75 [0.57, 0.98] | 0.94 [0.70, 1.27] |
| Prior use of lipid-lowering agents | – | – | 0.72 [0.59, 0.88] | 0.92 [0.74, 1.13] |
| Prior use of NSAIDS | 2.61 [1.50, 4.54] | 1.02 [0.57, 1.84] | – | – |

^a^In male patients, only 7 patients had dissociative, somatoform and adjustment disorders; therefore, they were combined with had also anxiety and mood disorders

List of abbreviations: aHR: Adjusted hazard ratios; CI: confidence interval; NSAIDS: nonsteroidal anti-inflammatory drugs

# **eTable 7: Sensitivity analysis – Predictors of switch to a TNFi/UST or add-on and CSA discontinuation among males and females with psoriasis – Without excluding patients with congestive heart failure (N=1,755)**

|  | Switch a TNFi/UST or add-on | | CSA discontinuation | |
| --- | --- | --- | --- | --- |
|  | Females (N=973) | Males (N=782) | Females (N=973) | Males (N=782) |
| Number of events | 66 | 62 | 595 | 487 |
|  | aHR (95% CI) | aHR (95% CI) | aHR (95% CI) | aHR (95% CI) |
| Cohort entry after 2011 | 1.80 [1.05, 3.07] | 1.42 [0.81, 2.49] | 0.82 [0.69, 0.97] | 0.74 [0.61, 0.90] |
| Age |  |  |  |  |
| 20-54 years | Ref | Ref | – | – |
| 55-64 years | 0.79 [0.44, 1.42] | 0.30 [0.15, 0.59] |  |  |
| 65-74 years | 0.65 [0.35, 1.20] | 0.22 [0.11, 0.47] |  |  |
| ≥75 years | 0.14 [0.03, 0.60] | 0.13 [0.04, 0.42] |  |  |
| Time to first CSA prescription |  |  |  |  |
| 0–2.99 months | Ref | Ref | – | – |
| 3­-12 months | 0.50 [0.24, 1.07] | 0.65 [0.22, 1.92] |  |  |
| >12 months | 0.75 [0.42, 1.34] | 1.96 [0.98, 3.92] |  |  |
| Specialty of the CSA prescriber |  |  |  |  |
| Dermatologist | – | – | Ref | Ref |
| Rheumatologist |  |  | 0.77 [0.58, 1.03] | 0.71 [0.51, 0.99] |
| Other specialists |  |  | 0.90 [0.71, 1.14] | 0.82 [0.62, 1.07] |
| First CSA received |  |  |  |  |
| Methotrexate | – | – | Ref | Ref |
| Cyclosporine |  |  | 2.05 [1.24, 3.39] | 1.15 [0.72, 1.84] |
| Acitretin |  |  | 2.08 [1.71, 2.52] | 1.65 [1.33, 2.04] |
| Sulfasalazine |  |  | 1.65 [1.09, 2.49] | 1.28 [0.82, 2.00] |
| Prior hospitalization | – | – | 0.99 [0.83, 1.17] | 0.73 [0.60, 0.89] |
| Rheumatoid arthritis | 0.46 [0.22, 0.95] | 1.25 [0.60, 2.58] | 0.72 [0.54, 0.94] | 0.84 [0.61, 1.18] |
| Obesity | 1.29 [0.50, 3.35] | 2.30 [0.89, 5.93] | – | – |
| Mental health disorders |  |  |  |  |
| No mental health disorder | Ref | Ref | – | – |
| Anxiety and mood disorders | 0.98 [0.54, 1.78] | 1.30 [0.72, 2.34] |  |  |
| Dissociative, somatoform and adjustment disorders | 3.17 [1.28, 7.83] | NA^a^ |  |  |
| Other mental health disorders | 1.42 [0.34, 5.98] | 1.41 [0.59, 3.39] |  |  |
| Prior use of Hypoglycemic agents | – | – | 0.80 [0.63, 1.02] | 0.98 [0.75, 1.27] |
| Prior use of lipid-lowering agents | – | – | 0.74 [0.62, 0.90] | 0.90 [0.74, 1.10] |
| Prior use of NSAIDS | 2.71 [1.58, 4.67] | 1.11 [0.64, 1.92] | – | – |

^a^In male patients, only 8 patients had dissociative, somatoform and adjustment disorders; therefore, they were combined with had also anxiety and mood disorders

List of abbreviations: aHR: Adjusted hazard ratios; CI: confidence interval; NSAIDS: nonsteroidal anti-inflammatory drugs

# **eTable 8: Sensitivity analysis – Predictors of switch to a TNFi/UST or add-on and CSA discontinuation among males and females with psoriasis – Grace period of 30 days (N=1,644)**

|  | Switch a TNFi/UST or add-on | | CSA discontinuation | |
| --- | --- | --- | --- | --- |
|  | **Females (N=916)** | **Males (N=728)** | **Females (N=916)** | **Males (N=728)** |
| Number of events | 59 | 40 | 607 | 496 |
|  | **aHR (95% CI)** | **aHR (95% CI)** | **aHR (95% CI)** | **aHR (95% CI)** |
| **Age** | 1.87 [1.07, 3.29] | 1.09 [0.54, 2.20] | 0.79 [0.66, 0.93] | 0.82 [0.68, 0.99] |
| 20-54 years | Ref | Ref | – | – |
| 55-64 years | 0.67 [0.36, 1.25] | 0.25 [0.10, 0.63] |  |  |
| 65-74 years | 0.61 [0.32, 1.16] | 0.17 [0.07, 0.46] |  |  |
| ≥75 years | 0.08 [0.01, 0.60] | 0.13 [0.03, 0.56] |  |  |
| **Time to first CSA prescription** |  |  |  |  |
| 0–2.99 months | Ref | Ref | – | – |
| 3­-12 months | 0.44 [0.19, 1.02] | 0.50 [0.13, 1.91] |  |  |
| >12 months | 0.78 [0.42, 1.42] | 1.38 [0.61, 3.08] |  |  |
| **Specialty of the CSA prescriber** |  |  |  |  |
| Dermatologist | – | – | Ref | Ref |
| Rheumatologist |  |  | 0.71 [0.53, 0.95] | 0.61 [0.43, 0.85] |
| Other specialists |  |  | 0.79 [0.62, 1.00] | 0.73 [0.56, 0.96] |
| **First CSA received** |  |  |  |  |
| Methotrexate | – | – | Ref | Ref |
| Cyclosporine |  |  | 2.23 [1.40, 3.56] | 0.85 [0.51, 1.43] |
| Acitretin |  |  | 1.99 [1.65, 2.42] | 1.47 [1.19, 1.80] |
| Sulfasalazine |  |  | 1.53 [1.01, 2.34] | 1.27 [0.80, 2.03] |
| **Prior hospitalization** | – | – | 0.98 [0.82, 1.17] | 0.76 [0.62, 0.93] |
| **Rheumatoid arthritis** | 0.43 [0.20, 0.93] | 1.48 [0.63, 3.48] | 0.73 [0.56, 0.97] | 0.93 [0.67, 1.30] |
| **Obesity** | 1.09 [0.37, 3.18] | 4.85 [1.60, 14.70] | – | – |
| **Mental health disorders** |  |  |  |  |
| No mental health disorder | Ref | Ref | – | – |
| Anxiety and mood disorders | 1.20 [0.65, 2.22] | 1.28 [0.59, 2.77] |  |  |
| Dissociative, somatoform and adjustment disorders | 3.32 [1.33, 8.30] | NA |  |  |
| Other mental health disorders | 2.35 [0.54, 10.32] | 1.93 [0.71, 5.23] |  |  |
| **Prior use of Hypoglycemic agents** | – | – | 0.81 [0.63, 1.03] | 0.91 [0.70, 1.20] |
| **Prior use of lipid-lowering agents** | – | – | 0.71 [0.59, 0.86] | 0.90 [0.74, 1.10] |
| **Prior use of NSAIDS** | 2.65 [1.47, 4.75] | 1.29 [0.64, 2.57] | – | – |

^a^In male patients, only 7 patients had dissociative, somatoform and adjustment disorders; therefore, they were combined with had also anxiety and mood disorders

List of abbreviations: aHR: Adjusted hazard ratios; CI: confidence interval; NSAIDS: nonsteroidal anti-inflammatory drugs

# **eTable 9: Sensitivity analysis – Predictors of switch to a TNFi/UST or add-on and CSA discontinuation among males and females with psoriasis – Grace period of 90 days (N=1,644)**

|  | Switch a TNFi/UST or add-on | | CSA discontinuation | |
| --- | --- | --- | --- | --- |
|  | **Females (N=916)** | **Males (N=728)** | **Females (N=916)** | **Males (N=728)** |
| Number of events | 69 | 63 | 507 | 403 |
|  | **aHR (95% CI)** | **aHR (95% CI)** | **aHR (95% CI)** | **aHR (95% CI)** |
| **Age** | 1.86 [1.11, 3.13] | 1.24 [0.71, 2.15] | 0.75 [0.62, 0.90] | 0.67 [0.54, 0.83] |
| 20-54 years | Ref | Ref | – | – |
| 55-64 years | 0.78 [0.45, 1.37] | 0.32 [0.16, 0.64] |  |  |
| 65-74 years | 0.54 [0.29, 1.00] | 0.20 [0.10, 0.42] |  |  |
| ≥75 years | 0.15 [0.03, 0.63] | 0.09 [0.02, 0.38] |  |  |
| **Time to first CSA prescription** |  |  |  |  |
| 0–2.99 months | Ref | Ref | – | – |
| 3­-12 months | 0.47 [0.22, 1.02] | 1.04 [0.39, 2.74] |  |  |
| >12 months | 0.85 [0.49, 1.49] | 2.02 [1.01, 4.05] |  |  |
| **Specialty of the CSA prescriber** |  |  |  |  |
| Dermatologist | – | – | Ref | Ref |
| Rheumatologist |  |  | 0.71 [0.52, 0.98] | 0.61 [0.42, 0.90] |
| Other specialists |  |  | 0.85 [0.65, 1.10] | 0.80 [0.59, 1.08] |
| **First CSA received** |  |  |  |  |
| Methotrexate | – | – | Ref | Ref |
| Cyclosporine |  |  | 2.17 [1.29, 3.65] | 1.14 [0.67, 1.94] |
| Acitretin |  |  | 1.93 [1.57, 2.38] | 1.62 [1.29, 2.04] |
| Sulfasalazine |  |  | 1.56 [0.98, 2.48] | 1.28 [0.76, 2.16] |
| **Prior hospitalization** | – | – | 1.04 [0.86, 1.27] | 0.73 [0.58, 0.92] |
| **Rheumatoid arthritis** | 0.44 [0.21, 0.91] | 1.25 [0.60, 2.60] | 0.70 [0.51, 0.95] | 0.95 [0.66, 1.36] |
| **Obesity** | 1.27 [0.49, 3.27] | 3.64 [1.25, 10.54] | – | – |
| **Mental health disorders** |  |  |  |  |
| No mental health disorder | Ref | Ref | – | – |
| Anxiety and mood disorders | 1.06 [0.61, 1.85] | 1.40 [0.78, 2.52] |  |  |
| Dissociative, somatoform and adjustment disorders | 3.05 [1.25, 7.48] | NA^a^ |  |  |
| Other mental health disorders | 1.51 [0.36, 6.34] | 1.77 [0.78, 4.02] |  |  |
| **Prior use of Hypoglycemic agents** | – | – | 0.77 [0.58, 1.02] | 0.92 [0.67, 1.25] |
| **Prior use of lipid-lowering agents** | – | – | 0.69 [0.56, 0.85] | 0.89 [0.72, 1.12] |
| **Prior use of NSAIDS** | 2.71 [1.60, 4.60] | 1.06 [0.61, 1.84] | – | – |

^a^In male patients, only 7 patients had dissociative, somatoform and adjustment disorders; therefore, they were combined with had also anxiety and mood disorders

List of abbreviations: aHR: Adjusted hazard ratios; CI: confidence interval; NSAIDS: nonsteroidal anti-inflammatory drugs

# **eTable 10: Sensitivity analysis – Predictors of switch to a biologic agent and CSA discontinuation among males and females with psoriasis – Without considering sulfasalazine as a CSA (N=1,610)**

|  | Switch a TNFi/UST or add-on | | CSA discontinuation | |
| --- | --- | --- | --- | --- |
|  | Females (N=901) | Males (N=709) | Females (N=901) | Males (N=709) |
| Number of events | 59 | 56 | 544 | 434 |
|  | aHR (95% CI) | aHR (95% CI) | aHR (95% CI) | aHR (95% CI) |
| Cohort entry after 2011 | 1.91 [1.08, 3.38] | 1.16 [0.65, 2.08] | 0.77 [0.64, 0.92] | 0.70 [0.57, 0.86] |
| Age |  |  |  |  |
| 20-54 years | Ref | Ref | – | – |
| 55-64 years | 0.81 [0.44, 1.48] | 0.29 [0.13, 0.61] |  |  |
| 65-74 years | 0.58 [0.30, 1.12] | 0.21 [0.09, 0.45] |  |  |
| ≥75 years | 0.08 [0.01, 0.61] | 0.09 [0.02, 0.39] |  |  |
| Time to first CSA prescription |  |  |  |  |
| 0–2.99 months | Ref | Ref | – | – |
| 3­-12 months | 0.43 [0.19, 0.97] | 1.95 [0.67, 5.68] |  |  |
| >12 months | 0.72 [0.39, 1.31] | 2.80 [1.18, 6.67] |  |  |
| Specialty of the CSA prescriber |  |  |  |  |
| Dermatologist | – | – | Ref | Ref |
| Rheumatologist |  |  | 0.77 [0.57, 1.05] | 0.63 [0.44, 0.90] |
| Other specialists |  |  | 0.96 [0.75, 1.23] | 0.88 [0.66, 1.17] |
| First CSA received |  |  |  |  |
| Methotrexate | – | – | Ref | Ref |
| Cyclosporine |  |  | 2.09 [1.26, 3.45] | 0.98 [0.58, 1.64] |
| Acitretin |  |  | 2.03 [1.66, 2.47] | 1.60 [1.29, 1.99] |
| Prior hospitalization | – | – | 0.96 [0.80, 1.16] | 0.71 [0.58, 0.89] |
| Rheumatoid arthritis | 0.54 [0.26, 1.13] | 1.62 [0.79, 3.34] | 0.65 [0.48, 0.89] | 0.85 [0.59, 1.22] |
| Obesity | 0.80 [0.24, 2.65] | 3.23 [1.11, 9.39] | – | – |
| Mental health disorders |  |  |  |  |
| No mental health disorder | Ref | Ref | – | – |
| Anxiety and mood disorders | 1.16 [0.63, 2.14] | 1.46 [0.80, 2.67] |  |  |
| Dissociative, somatoform and adjustment disorders | 3.48 [1.39, 8.69] | NA^a^ |  |  |
| Other mental health disorders | 1.68 [0.40, 7.13] | 1.56 [0.64, 3.81] |  |  |
| Prior use of Hypoglycemic agents | – | – | 0.74 [0.57, 0.97] | 1.06 [0.79, 1.41] |
| Prior use of lipid-lowering agents | – | – | 0.70 [0.58, 0.86] | 0.82 [0.66, 1.02] |
| Prior use of NSAIDS | 2.61 [1.47, 4.61] | 1.06 [0.58, 1.90] | – | – |

^a^In male patients, only 6 patients had dissociative, somatoform and adjustment disorders; therefore, they were combined with had also anxiety and mood disorders

List of abbreviations: aHR: Adjusted hazard ratios; CI: confidence interval; NSAIDS: nonsteroidal anti-inflammatory drugs

# **eFigure 1: Study flowchart**

Patients ages ≥20 years with a ≥1 psoriasis diagnosis between January 01, 2002 and September 30, 2015

**N = 33,108**

**Excluded N = 29,155**

With <1 year of enrollment in the provincial drug plan in the year prior to the first psoriasis diagnosis **N = 8,929**

Prior use of phototherapy and systemic agents (biologics or CSA) in the year prior to the first psoriasis diagnosis **N = 2,227**

Without a prescription fill of a CSA during the follow-up **N= 17,999**

Individuals with a CSA prescription fill

**N = 1,773**

**Excluded N = 129**

With <3 months of follow-up **N = 80**

With a prescription fill for a biologic prior to receiving a CSA **N = 13**

With a diagnosis of HIV, HBV, tuberculosis, congestive heart failure and melanoma skin cancer in the two years prior to the first CSA prescription fill **N = 33**

Received a combination of CSA **N = 3**

Study cohort

**N = 1,644**

Patients with a first psoriasis diagnosis

**N = 30,928**

**Excluded N = 2,180**

With a psoriasis diagnosis in the prior three years **N = 2,180**

**List of abbreviations:** CSA: conventional systemic agent; HBV: Hepatitis B virus, HIV: Human immunodeficiency virus

# **eFigure 2: Kaplan Meier estimates of switch to (a) TNFi/UST or add-on; and (b) CSA discontinuation by sex**

**(a)**

**(b)**

**List of abbreviations:** CSA: Conventional systemic agents; TNFi/UST: Tumor necrosis factor inhibitors and ustekinumab
